# Effect of Submucosal Cryotherapy on Postoperative Pain in Maxillary Premolars with Symptomatic Irreversible Pulpitis: A Prospective, Parallel, Triple-Blinded Randomized Controlled Trial

**DOI:** 10.1055/s-0045-1806934

**Published:** 2025-05-30

**Authors:** Mai Shalabi, Abeer H. Mahran, Tarek Elsewify

**Affiliations:** 1 Department of Endodontic, Faculty of Dentistry, Ain Shams University, Cairo, Egypt; 2 Department of Restorative Dental Sciences, College of Dentistry, Gulf Medical University, Ajman, United Arab Emirates

**Keywords:** analgesics, cryotherapy, inflammation, postoperative pain

## Abstract

**Objective:**

To compare the effect of submucosal cryotherapy with steroids and nonsteroidal injections on postendodontic pain in maxillary premolars with symptomatic irreversible pulpitis.

**Materials and Methods:**

A total of 52 patients with maxillary premolars diagnosed with symptomatic irreversible pulpitis were randomly allocated into four equal groups ( *n* = 13). Cold saline was injected submucosally in the experimental groups, while the two active comparator groups received dexamethasone sodium phosphate and diclofenac sodium submucosally. The control group did not receive any injections. Preoperative pain level was recorded using a numerical pain scale and subtracted from the postoperative pain level at 6, 24, 48, and 72 hours, to calculate the pain reduction, after single-visit root canal treatment. Data were statistically analyzed at a significance level of *p* < 0.05.

**Results:**

Pain score reduction did not differ significantly between all the groups at all intervals. All groups showed a significant increase in pain reduction with time. The control and cryotherapy groups showed significantly lower incidence of flare-ups than the diclofenac group.

**Conclusion:**

Submucosal cryotherapy can be used as a safe and conservative alternative to steroids and nonsteroidal anti-inflammatory drugs (NSAIDs) in the management of postoperative pain in cases with symptomatic irreversible pulpitis.

**Clinical Relevance:**

Submucosal cryotherapy reduces postoperative endodontic pain and can be used as a safe and conservative alternative to steroid and NSAID injections for postoperative endodontic pain control in cases with symptomatic irreversible pulpitis.

## Introduction

Postoperative pain is a common sequela after root canal treatment. Postoperative pain ranges from mild to severe and typically manifests within a few hours to a few days after the procedure. It is more likely in teeth with substantial underlying inflammation or infection. 1

Mechanical factors, such as over-instrumentation, can contribute to postoperative pain by causing the enlargement of the apical foramen. This excessive pressure during treatment can lead to debris extrusion, where microorganisms and irritating materials are pushed beyond the root apex, resulting in discomfort and inflammation. 2 3 Chemical factors also play a role; certain irrigating solutions and medicaments used during root canal therapy can irritate surrounding tissues if they extend beyond the apex and are not properly rinsed out. 4 5 6

Understanding these etiologies is crucial in managing and minimizing postoperative pain for patients undergoing root canal treatment. Several strategies have been presented in endodontics for managing postoperative pain such as using long-lasting anesthesia 7 and prescribing analgesics such as nonsteroidal anti-inflammatory drugs (NSAIDs), acetaminophen, and corticosteroids. 8 9 10 Although they are relatively safe medications, they have been linked to gastrointestinal intolerance, as well as renal, hepatic, and respiratory disorders. 11 12

Cryotherapy is a relatively new and conservative method of pain control that has gained attention over the past decade as an effective adjunct for managing postoperative pain. This treatment involves exposing a specific body area to cold temperatures, aiming to lower tissue temperature to promote healing and achieve other therapeutic effects such as reducing edema, inflammation, and pain. In dentistry, cryotherapy has been effective in reducing pain following periodontal surgeries, extractions, and implant procedures. In 2015, intracanal cryotherapy was introduced in endodontics and has proven successful in alleviating postoperative endodontic pain. 13 Several studies demonstrated a significant reduction in postoperative pain after cryotherapy when used as a final irrigating solution. 14 15 16 17 A recent study introduced the novel approach of applying cold saline solution to the submucosal tissues surrounding the tooth. 18

NSAIDs, such as diclofenac sodium, inhibit the activity of the cyclooxygenase-2 enzyme, which is highly expressed in cases of irreversible pulpitis. This inhibition reduces the production of prostaglandins (PGs) and proinflammatory cytokines, 19 reducing postoperative endodontic pain. 20

Glucocorticosteroids, such as dexamethasone sodium phosphate, can mitigate acute inflammation by reducing vasodilation and neutrophil migration. They also block the inflammatory cascade by inhibiting the phospholipase enzyme, reducing the formation of arachidonic acid and its metabolites. This results in a subsequent decrease in the production of PGs and leukotrienes. 21

The submucosal route of application of cryotherapy is a novel approach that has not been tested clinically before for the management of postoperative endodontic pain. Based on the results of the animal study by Shalabi et al, 18 it was deemed important to conduct a randomized clinical trial to evaluate this route of administration of cryotherapy, as a safe and conservative method, and compare it to other anti-inflammatory drugs in the management of postendodontic pain.

No difference between the cold saline application, and the steroids and nonsteroids on the postoperative endodontic pain level in cases of symptomatic irreversible pulpitis in maxillary premolars is the null hypothesis tested in the current study.

## Materials and Methods

### Ethical Considerations

**Fig. 1:**
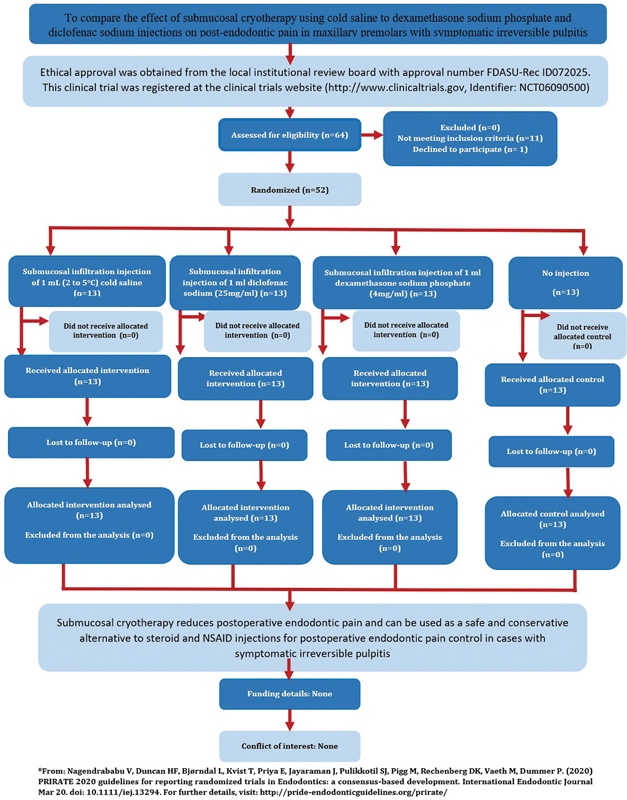
The PRIRATE 2020 flowchart for reporting clinical trials.

Ethical approval was obtained from the local Institutional Review Board with approval number FDASU-Rec ID072025. This clinical trial was registered at the clinical trials website ( https://www.clinicaltrials.gov , identifier: NCT06090500). Patients were treated according to the principles of the World Medical Association Declaration of Helsinki. 22 Reporting of this study was done following the PRIRATE 2020 guidelines ( Supplementary Material , available in the online version), 23 as demonstrated in Fig. 1 .

### Trial Design and Sample Size Calculation

This study was designed as a prospective, parallel, triple-blind phase IV randomized clinical trial. A power analysis was performed based on the results of Yavari et al. 24 Using an alpha (α) level of 0.05 (5%) and power = 80%, the predicted sample size ( *n* ) is a total of 52 cases, that is, 13 cases per group. Sample size calculation was performed using the sample size calculator at www.clinical.com.

### Eligibility Criteria

The participants in this trial were allocated from the outpatients' emergency clinic of the endodontic department during an 8-month period between May 2023 and January 2024. Inclusion criteria were the American Society of Anesthesiologists class I complaint patients with an age range of 18 to 50 years suffering from symptomatic irreversible pulpitis with normal apical tissues of a double-canaled maxillary premolar. Patients who reported allergy to any of the drugs tested in the study, anti-inflammatory intake, or long-term steroid therapy were excluded. Any procedural mishap during the root canal treatment also excludes the patient from the study.

All participants were informed about the benefits, risks, and possible side effects of the proposed interventions. An informed consent of the treatment was signed by each patient.

### Randomization and Blinding

A total of 52 patients who fulfilled the eligibility criteria were randomly allocated according to the ratio of 1:1:1:1 to each of the four treatment groups. Randomization was performed by using computer-generated randomization software ( www.sealedenvelope.com ).

#### Allocation Concealment

Two independent individuals were responsible for the random sequence generation and allocation concealment before the initiation of the study. One of them had to withhold the allocation sequence from the investigators in an opaque and tightly sealed envelope to avoid selection bias in the recruitment stage. The other individual was responsible for the injection of the assigned solution in the randomized sequence by filling the insulin syringe with the required medication/saline preoperatively and discarding the ampoule out of sight from the operator responsible for the treatment to keep the operator blinded.

#### Implementation

Before commencing the endodontic treatment, preoperative submucosal infiltration injections were assigned by telephone verification from the first individual (withholding the allocation sequence) to the second one (responsible for the injection), to inform them which solution should be injected. The operator is kept blinded the whole time and is responsible only for the endodontic treatment. Consequently, participants were enrolled into each of the four study groups, and all individuals in the experimental groups were injected with different prescriptions. An experienced endodontist, blinded to the study, was responsible for the pain assessment and data collection. Thus, this study was triple-blinded. The biostatistician was also blinded to the whole study.

### Patients' Classification

All patients were presented with a history of diffuse, spontaneous moderate-to-severe lingering pain, simulated by cold or hot stimuli, and stayed for several minutes after removal of stimulation. Periapical tissues were clinically normal with no tenderness with percussion or palpation. Pulp vitality was then confirmed clinically by directly observing hemorrhage inside the canals. Techniques of instrumentation and obturation were standardized for all the patients.

A total of 65 patients were recruited and evaluated for the eligibility criteria. Thirteen patients were excluded either due to not falling within the inclusion criteria ( *n* = 11) or rejection of participation in the study ( *n* = 1). A total of 52 patients applied to the inclusion criteria and were allocated to this clinical trial. They received the submucosal injections (except for the control group) and were then analyzed for the level of pain experienced postoperatively.

They were randomly allocated into four groups ( *n* = 13) according to the submucosal injection solution used, as follows:

*Diclofenac group* : 1 mL of diclofenac sodium (25 mg/mL).
*Dexamethasone group* : 1 mL of dexamethasone sodium phosphate (4 mg/mL).
*Cryotherapy group* : 1 mL of cold saline at a temperature of 2 to 5°C.
*Control group* : No injection other than the local anesthetic solution was administered.

### Procedural Steps

After signing an informed consent of the treatment, the patient's demographic data were recorded, and preoperative radiographs were exposed using an intraoral digital imaging system (Fona ScaNeo, Assago, Italy).

A numeric pain rating scale (NRS), with numbers ranging from 0 indicating no pain up to 10 indicating the worst possible pain 26 27 was utilized to record the level of preoperative pain by a blinded trained clinician who explained it to the patient.

Teeth were anesthetized by a single carpule (1.7 mL) of local anesthetic solution (4% articaine with 1:100,000 epinephrine) using an infiltration injection technique in the depth of the vestibule apical to the tooth to be treated.

All caries or defective restorations were then removed, and an access cavity was prepared using a tapered diamond stone with an air-driven high-speed handpiece under copious water coolant.

After achieving appropriate isolation using a rubber dam, negotiation of the root canals was then performed first by maintaining apical patency using K-files #10 and #15 (Mani Inc., Tochigi, Japan), working length determination was then determined using Root ZX electronic apex locator (Morita Corporation, Kyoto, Japan) and confirmed with periapical radiographs.

M-Pro rotary file system (Innovative Material and Devices, Inc, Shanghai, China) was used for canal preparation with torque and speed adjusted according to the manufacturer's instructions followed by enlarging the apical diameter of each canal till K-flex file #35 (Mani Inc.). Copious irrigation during preparation with 5.25% sodium hypochlorite was done using a 30-G side vented needle (Fanta Dental Materials Co., Ltd, Shanghai, China) placed 1 mm short of the apex with an in-and-out activating motion. Regular confirmation of patency followed by irrigation was performed between the enlarging instruments.

After complete canal preparation, obturation was performed in the same visit using gutta-percha and resin-based root canal sealer by the cold lateral compaction technique. To achieve a proper coronal seal, the access cavity was immediately restored with at least 4-mm-thick Coltosol F (Coltene Whaledent, Altstatten, Switzerland).

Patients were instructed to record the presence and severity of pain experienced postoperatively at different time intervals guided by the given pain scale explained earlier. They were asked to report the pain level at the first 6, 24, 48, and 72 hours following root canal obturation. 28 The preoperative pain level was subtracted from the pain score at each interval to calculate the reduction in pain level at each interval. The presence or absence of postoperative flare-ups in the form of intra- or extraoral swelling was also recorded. The filled data were then collected from all patients by a blinded trained clinician for analysis. During the study period, patients were instructed not to take analgesics and to report if any were taken for exclusion of the patient. Safety monitoring was performed throughout the follow-up period based on any adverse events reported by the patients on their initiative.

### Statistical Analysis

Fisher's exact test was applied to the categorical data. Numerical data were checked for normality using Shapiro–Wilk's test. One-way analysis of variance was applied to the normally distributed age data. Kruskal–Wallis' test for intergroup comparisons and Friedman's test, followed by Nemenyi's post hoc test for intragroup comparisons were applied for the nonnormally distributed pain score values. The *p* -values were adjusted for multiple comparisons using Bonferroni correction. The significance level was set at ( *p* < 0.05) within all tests. Statistical analysis was performed with R statistical analysis software version 4.3.3 for Windows (R Foundation for Statistical Computing, Vienna, Austria).

## Results

### Demographic Data

Intergroup comparisons and summary statistics for demographic data are presented in Table 1 . There was no significant difference between tested groups regarding gender distribution ( *p* = 0.126), age ( *p* = 0.755), treated teeth ( *p* = 0.907), and preoperative pain score ( *p* = 0.669). The mean age for the control group was 32.38 ± 6.32, the diclofenac group was 30.88 ± 9.49, the dexamethasone group was 36.00 ± 11.49, and the cryotherapy group was 32.60 ± 9.41 years. The mean of the preoperative pain score was 8.31 ± 2.02, 8.46 ± 1.45, 8.46 ± 1.51, and 9.08 ± 1.26, respectively.

**Table 1:** Intergroup comparisons and summary statistics for demographic data

Parameter		Control	Diclofenac	Dexamethasone	Cryotherapy	*p* -Value
Gender, *n* (%)	Male	2 (15.38%)	7 (53.85%)	2 (15.38%)	3 (23.08%)	**0.126**
	Female	11 (84.62%)	6 (46.15%)	11 (84.62%)	10 (76.92%)	
Age, y (mean ± SD)		32.38 ± 6.32	30.88 ± 9.49	36 ± 11.49	32.60 ± 9.41	**0.755**
Tooth, *n* (%)	First premolar	9 (69.23%)	8 (61.54%)	10 (76.92%)	10 (76.92%)	**0.907**
	Second premolar	4 (30.77%)	5 (38.46%)	3 (23.08%)	3 (23.08%)	
Preoperative pain score (mean ± SD)		8.31 ± 2.02	8.46 ± 1.45	8.46 ± 1.51	9.08 ± 1.26	**0.669**

Abbreviation: SD, standard deviation.

Note: Value in bold indicates nonsignificant ( *p* > 0.05).

### Pain Score Reduction

The mean and median of reduction in pain score are detailed in Table 2 with the inter- and intragroup comparisons. The cryotherapy group showed the highest reduction in the pain score; yet, the difference was not statistically significant at all intervals ( *p* > 0.05). Within all groups, there was a significant increase in pain reduction with time ( *p* < 0.05).

**Table 2:** Inter-, intragroup comparisons, mean, SD, and median values for reduction in pain score

Time	Measurement	Control	Diclofenac	Dexamethasone	Cryotherapy	*p* -Value
6 h	Mean ± SD	4.23 ± 3.27 ^Ac^	4.46 ± 3.18 ^Ac^	5.69 ± 2.78 ^Ab^	6.00 ± 2.92 ^Ab^	**0.406ns**
	Median (IQR)	3.25 (6) ^Ac^	5.25 (6) ^Ac^	5.67 (4) ^Ab^	6.00 (5) ^Ab^	
24 h	Mean ± SD	5.77 ± 2.92 ^Ab^	6.00 ± 2.97 ^Ab^	5.46 ± 3.55 ^Ab^	6.92 ± 3.23 ^Aab^	**0.644ns**
	Median (IQR)	5.75 (4) ^Ab^	7.00 (5) ^Ab^	7.85 (5) ^Ab^	7.88 (3) ^Aab^	
48 h	Mean ± SD	7.46 ± 2.30 ^Aa^	6.62 ± 2.90 ^Aab^	6.85 ± 2.38 ^Aab^	7.77 ± 2.49 ^Aa^	**0.621ns**
	Median (IQR)	7.75 (4) ^Aa^	7.67 (6) ^Aab^	7.25 (2) ^Aab^	8.33 (3) ^Aa^	
72 h	Mean ± SD	7.92 ± 2.14 ^Aa^	7.31 ± 2.53 ^Aa^	7.77 ± 1.92 ^Aa^	8.31 ± 2.06 ^Aa^	**0.743ns**
	Median (IQR)	8 (4) ^Aa^	7.88 (4) ^Aa^	7.75 (4) ^Aa^	8.75 (2) ^Aa^	
*p* -Value		**<0.001**	<0.001*	0.003*	<0.001*	

Abbreviations: IQR, interquartile range, SD, standard deviation.

Notes: Values with different upper and lowercase superscript letters within the same horizontal row and vertical column, respectively, are significantly different; *, significant ( *p* < 0.05); ns, nonsignificant ( *p* > 0.05).

### Flare-up Incidence

Intergroup comparison, frequency, and percentage values for flare-up incidence are presented in Table 3 . In the diclofenac group, flare-up occurred in six cases, and in dexamethasone, it occurred in three cases, while other groups were free. The difference in incidence was statistically significant with the control group and cryotherapy having significantly lower incidence than the diclofenac group ( *p* = 0.003).

**Table 3:** Intergroup comparison, frequency, and percentage values for flare-up incidence

Flare-up	*n* (%)				*p* -Value
	Control	Diclofenac	Dexamethasone	Cryotherapy	
No	13 (100%) ^A^	7 (53.85%) ^B^	10 (76.92%) ^AB^	13 (100%) ^A^	0.003*
Yes	0 (0%) ^A^	6 (46.15%) ^B^	3 (23.08%) ^AB^	0 (0%) ^A^	

Note: Values with different superscript letters within the same horizontal row are significantly different; *, significant ( *p* < 0.05); ns, nonsignificant ( *p* > 0.05).

## Discussion

Proper management of postoperative endodontic pain is deemed mandatory to deliver a standard of care to the patient. 29 Premedication with NSAIDs, 21 steroids, 21 30 and opioids 30 have been previously advocated to be used to control postoperative endodontic pain levels, especially in cases of symptomatic irreversible pulpitis.

Although steroids and nonsteroidals are reported to successfully manage postoperative endodontic pain due to their ability to block the inflammatory cascade at different points, 31 32 33 steroids are not recommended due to their well-known systemic side effects. NSAID also shows fewer side effects and has some precautions during prescription which raises the point of looking for a safer and more conservative alternative.

Cryotherapy emerged in the past few years as a potent and safe alternative for the management of endodontic pain. The most popular way of applying cryotherapy and lowering the tissue temperature is by applying a cold irrigant inside the root canal space following the root canal preparation. 34 35 Another familiar approach is following endodontic microsurgery, through the application of cold packs. 36 37 However, no clinical trials have been conducted till now to assess the submucosal injection route of cryotherapy in endodontics. This is the first clinical trial to assess this route in maxillary premolars diagnosed with symptomatic irreversible pulpitis and normal apical tissues.

Based on the results of the animal study by Shalabi et al, 18 it was deemed important to conduct a randomized clinical trial to compare the effects of submucosal cryotherapy and submucosal anti-inflammatory drug injections on postoperative pain experience.

Submucosal cryotherapy has the potential to enhance patient comfort, reduce reliance on systemic medications, and minimize adverse effects. This approach can provide more effective and localized analgesia. The integration of this novel technique into routine dental practice may lead to standardized protocols, improving pain management outcomes while fostering patient trust and compliance. With further research and refinement, this approach could become a cornerstone in evidence-based dentistry, aligning with the broader goals of precision medicine and patient-centered care.

In the current study, patients chosen to be enrolled were those diagnosed with symptomatic irreversible pulpitis. According to Segura-Egea et al 38 and Gotler et al, 39 the severity of pain experienced following root canal treatment was significantly higher in individuals presented with vital pulps in comparison to necrotic ones.

Maxillary teeth were selected for this clinical trial. The maxilla is formed mainly of cancellous bone with large medullary spaces and with no or thin, porous cortical plate allowing for better diffusion of the injected agents to better reach the apical and periapical areas of the target teeth. Maxillary premolars were selected because of the decreased risk of errors or missed canals during mechanical preparation in comparison to maxillary molars. They also provide a relatively larger volume of pulp tissue than maxillary anterior teeth and thus an expected larger amount of inflammatory mediators released. Therefore, maxillary premolars were thought to be a suitable choice for the current study. For standardization, only premolars with double canals were included in this clinical trial.

Taking into consideration the results reported by Patel et al 40 demonstrating different outcomes of endodontic treatment for different racial groups, all patients included in the study were of the same ethnic background to avoid any inconsistency in recording the posttreatment data.

For anesthesia, 4% articaine hydrochloride, 1:100,000 adrenaline was the solution of choice. Nagendrababu et al 41 demonstrated that in cases diagnosed with symptomatic irreversible pulpitis, articaine was able to exhibit greater efficacy in comparison to lidocaine. Only one anesthetic cartridge was administered for standardization and to avoid the effect of different anesthetic volumes on the results. Patients who needed extra administration of an anesthetic solution for pain control were excluded from the study.

Single-visit root canal treatment was performed to overcome the drawback of noncompliant patients, failure of recall, and to eliminate the effect of different numbers of treatment visits on the results. Joshi et al 42 and Izadpanah et al 43 reported a statistically significant increase in postoperative pain levels recorded in single-visit endodontic treatment.

It was reported by Karcioglu et al 44 and Alghadir et al 45 that all pain scales were proven to be valid, reliable, and suitable to be utilized in clinical research work. In the current trial, pain assessment was accomplished via a unidirectional pain scale such as the NRS, which exhibits a good level of sensitivity and provides analyzable data. Pain, however, is a completely subjective feeling imposing a challenge on precise data collection.

No statistically significant difference in the pain score reduction was noted between all the groups at all time intervals which leads to acceptance of the null hypothesis. All groups, experimental and control groups, showed a significant reduction in pain scores along all of the intervals. This could be explained by the natural uptake of the proinflammatory mediators released within the tissues and the start of the healing phase after cessation of active inflammation in addition to the role of the anti-inflammatory mediators. Emad et al 17 showed downregulation of interleukin (IL)-1β gene expression and a significant upregulation of IL-10 on the seventh day postoperatively. Cryotherapy results using the intracanal approach yielded zero pain scores after 1 week. 46 47 48 These results are in agreement with those reported by Yavari et al 24 and Aksoy and Ege. 30

Cryotherapy showed the greatest reduction in pain levels, although not statistically significant. This could be related to the decreased vascularity in the periapical area due to the submucosal application of the cold saline which will lead to a reduction in tissue temperature, decreased edema, decreased oxygen consumption, reduced free radicals release, reduced level of tissue damage, and affecting the nerve conduction capacity of nociceptors and thermoreceptors. 18

These findings are consistent with the findings reported by Shalabi et al. 18 Their animal study showed a reduction in substance P and IL-6 release with cryotherapy in comparison to dexamethasone and diclofenac groups. Direct comparison to other clinical studies is not applicable, as none has utilized the submucosal approach of cryotherapy before. Meanwhile, our results come in agreement with Shah et al, 49 Bazaid and Kenawi, 35 and Al-Nahlawi et al 25 who showed a significant reduction in pain scores using intracanal cryotherapy.

This successful pain reduction could be compared to those reported by Keskin et al 50 who advocated intracanal cryotherapy and showed a reduction in pain scores in all tested groups at 72 hours following treatment that was statistically significant except for the 24-hour interval. The difference in significance could be due to the difference in the approach of the cryotherapy application, as well as different teeth selection, and preoperative status. However, another important factor to be considered is that our results in this randomized controlled trial showed a nonparametric distribution of the data. Nonparametric results could be an indication that a larger sample size recruitment could result in a rather statistically significant difference. Another point that could account for this is transforming the pain scale reading to numeric data, starting with a high pain level and then ending with almost a zero, which yields a very large standard deviation and abnormally distributed data.

On the contrary, Alharthi et al 51 and Akpinar et al 52 evaluated the intracanal cryotherapy and showed no significant difference compared with the control one. This could be attributed to the asymptotic cases studied by Alharthi et al and the two-visit approach with intracanal medication placed by Akpinar et al.

The higher incidence of flare-ups in the dexamethasone and diclofenac groups could be explained by the fact that anti-inflammatory medications such as diclofenac and dexamethasone act by interrupting the inflammatory cascade, either at the cyclooxygenase enzyme level or at the phospholipase enzyme level, respectively. These actions, although offer a beneficial effect regarding the control of the inflammatory process and pain perception via reducing the level of mediators released, could also result in hindering the healing mechanisms generated at the final phases of the inflammatory cascade by various cytokines, leukotrienes, and PGs. This interruption could offer a reasonable explanation for the increased incidence of flare-ups due to affecting the healing process.

It was reported in the literature by Lisowska et al, 53 in their review studying the impact of NSAIDs on healing, that the negative effect of these drugs on the healing process could be mainly related to COX-2 blockade. They also reported an inhibitory effect of NSAIDs on angiogenesis as confirmed by Murnaghan et al, 54 which could result in delayed healing. The review also mentioned that retrospective studies advocating large groups of patients reported that long-term use of NSAIDs multiplies the risk of developing healing disorders. The negative impact of corticosteroids on the healing process was also reported in the literature due to their interference with inflammation, angiogenesis, fibroblast proliferation, and collagen synthesis. 55

Limitations of the current study include using the NRS, which is a subjective method of pain evaluation. The newly provided objective methods for pain assessment such as electroencephalography, heart rate variability, and electromyography are recommended for future research work in combination with the basic pain scale methods. A larger sample size and/or a novel statistical manipulation of pain scores are recommended in future pain postoperative assessment studies. Further evaluation of the efficiency of submucosal cryotherapy is recommended across mandibular molars and premolars, as well as across different patient demographics. The combination of submucosal cryotherapy with other traditional pain management techniques is also recommended.

## Conclusion

Submucosal cryotherapy reduces postoperative endodontic pain and can be used as a safe and conservative alternative to steroid and NSAID injections for postoperative endodontic pain control in cases with symptomatic irreversible pulpitis.
