# Supplementary material for: Effect of Submucosal Cryotherapy on Postoperative Pain in Maxillary Premolars with Symptomatic Irreversible Pulpitis: A Prospective, Parallel, Triple-Blinded Randomized Controlled Trial
Source: Eur J Dent. 2025 May 30;20(1):236–44. doi: 10.1055/s-0045-1806934 (PMC12890412; doi:10.1055/s-0045-1806934)
Supplement: Supplementary file 1 — Supplementary Material [file 10-1055-s-0045-1806934-s24124013.pdf]

Supplementary Material PRIRATE 2020

Checklist of items to be included when reporting randomized trials in endodontics[a]

Section/topic	Item number	Checklist items	Reported on page number
Title	1a	The phrase "randomized clinical trial" or "randomized controlled trial" must be included in the title	1
	1b	Details of the specific area(s) of interest using words and phrases that identify the clinical problem and the intervention(s) must be provided	1
Keywords	2a	Keywords indicating the specific area(s) of interest using MeSH terms must be included	2
Abstract	3a	The Introduction of the abstract must explain briefly the rationale for the trial	1
	3b	The aim/objective(s) of the trial must be provided at the end of the Introduction section within the abstract	1
	3c	The Methodology section within the abstract must provide essential information on the nature of the trial (e.g., superiority, noninferiority, equivalence), its design (e.g., parallel, split-mouth, crossover), the inclusion/exclusion criteria, randomization process, blinding process, and statistical analysis	1
	3d	The Results section within the abstract must describe the number of participants that were randomized and analyzed, the size and direction (group favored) of the difference(s) between the intervention(s) and control groups with statistical analysis (*p*-values and 95% CI). Adverse events or side effects (if any) must also be reported or if none occurred, that must be mentioned explicitly	1
	3e	The Conclusion section within the abstract must summarize the findings and emphasize the clinical implication(s) of the results	2
	3f	The prospective registration (number and name of the registry) and source(s) of funding must be provided	2
Introduction	4a	The scientific background and rationale for the trial must be provided, including the gap(s) or inconsistencies in knowledge	3 and 4
	4b	The specific aim/objective(s) of the trial must be provided and the main clinical research question must be formulated clearly, preferably using the PICO framework (Problem/Population, Intervention, Control, and Outcome)	4
Methods<br>*Trial design*	5a	Details of the nature of the trial (superiority, noninferiority, equivalence of experimental intervention[s]), its design (parallel, split-mouth, crossover, single/double blinded) and test:control allocation ratio must be provided. If applicable, important information about the study design must also be provided, e.g., pragmatic or preference trial, phase (drug trials), patient or public involvement in planning, etc.	5
	5b	Changes to the methodology after the trial commenced (such as eligibility criteria) must be provided along with detailed explanations	6 and 7
a priori protocol	5c	Details of the ethical approval of the protocol and the process for obtaining informed consent must be provided	5
	5d	Details of the trial protocol including registration number and name of registry/clinical database and where it can be accessed (open access webpage, if applicable) must be provided	5
Participants (patients, operators, evaluators)	5e	A list of inclusion and exclusion criteria at the individual/tooth/root level must be provided	6 and 7
	5f	Details of the setting/environment of the trial must be provided. Details on how many operators were involved in performing the intervention and control and their relevant experience/qualifications are essential. The setting where the data	6–9

 © 2025. The Author(s).

(*Continued*)

Section/topic	Item number	Checklist items	Reported on page number
		were collected must be described. If several operators are included and/or if it is a multicenter setup, details of how standardization/calibration between individuals or centers were achieved must be provided	
	5g	The treatments in the intervention (experimental) group(s) must be described with sufficient detail to allow replication, including how and when they were actually administered	9–13
	5h	The intervention(s) or absence of intervention(s) in the control group must be described with sufficient details to allow replication, including how and when the intervention(s) was actually administered	9–13
Outcomes measures	5i	The primary and secondary (if any) outcome measures must be described, including how and when they were assessed and by whom	12 and 13
	5j	Details of any changes made to the study outcomes after the commencement of the trial must be described	9–13
	5k	If primary or secondary outcomes are to be regarded as surrogate outcomes, the rationale and empirical support for the connection between surrogate(s) and the outcome(s) of clinical relevance must be provided	12 and 13
Sample size	5l	How the sample size was determined must be described with reference to the published literature or a pilot study. The sample size may be modified after an internal feasibility study. Sample size calculations should generally refer to the primary outcome measure. If secondary outcome measures constitute the base for sample size calculation, an explanation must be provided	6
	5m	Any interim analyses and stopping guidelines must be described, when applicable	12 and 13
Randomization and allocation concealment	5n	The method used to generate the random allocation sequence along with any details of the type of restriction (e.g., blocking) if applicable must be described. The persons responsible for randomization and recruitment must be provided. For multicenter trials, a central randomization procedure is preferred and must be described. The unit of randomization should be specified and justified. Any stratification variables must be detailed	
	5o	Methods for allocation concealment up to the assignment of the participants into the intervention groups must be described	8 and 9
Blinding	5p	Information on who was/were blinded after assignment to the interventions (e.g., participants, caregivers, evaluators) must be described in detail. Blinding through masking of interventions (e.g., similar looking drugs/instruments) should be described. Detailed reasons for lack of blinding (if applicable) must be described	8 and 9
Statistical analysis	5q	The statistical methods used for analysis of the primary and secondary (if any) outcomes, additional subgroup analyses and adjusted analyses (if applicable) must be described in detail. Consideration of dropouts should be included in the calculations	13
	5r	How any cluster effects were managed during the analysis must be described	
Results	6a	The number of participants who were randomly assigned, received the intended treatment, and were analyzed for the primary and secondary (if any) outcome(s) for each group must be described. A flowchart must be provided	14–17
	6b	Reasons for losses/dropouts and exclusions after randomization must be described for each group and included in the flowchart. If intention-to-treat analyses are used, details of the process must be provided	14–17

(*Continued*)

 © 2025. The Author(s).

(*Continued*)

Section/topic	Item number	Checklist items	Reported on page number
	6c	The dates of recruitment, follow-up, and study duration must be described	14–17
	6d	Reason(s) for any early termination of the trial must be described	14–17
	6e	The baseline demographic and clinical characteristics of each group must be provided	14–17
	6f	The results for each group for each primary and secondary (if any) outcome(s), along with the estimated effect size and its precision, must be provided	14–17
	6g	Both absolute and relative effect sizes for binary outcomes must be provided	14–17
	6h	The results from any other analyses performed must be described, including subgroup analyses and adjusted analyses, distinguishing prespecified from exploratory	14–17
	6i	The incidence and management of any adverse effects or unintended effects in each group must be described	14–17
Discussion	7a	An estimate of the overall internal validity must be provided as well as the generalizability (external validity, applicability, real-world relevance) of the trial findings	17–19
	7b	The rationale for inclusion, exclusion criteria and study duration must be provided	18–20
	7c	An explanation of the clinical relevance of the primary and secondary outcomes must be provided	22 and 23
	7d	A detailed interpretation consistent with results, balancing benefits and harms, and considering other relevant evidence must be provided	19–23
	7e	The strength(s) of the trial must be provided	19–23
	7f	The limitations of the study must be provided, addressing the sources of potential bias, imprecision and, if applicable, multiplicity of analyses	23
	7g	Implication for future research and clinical practice must be described	23
Conclusion	8a	A rationale for the conclusion(s) must be provided, and the clinical significance must be highlighted	23
	8b	Explicit conclusion(s) from the trial must be provided	23
Funding details	9a	Sources of funding and other support (such as supply of drugs, equipment) as well as the role of funders must be acknowledged and described	1
Conflict of interest	10a	An explicit statement on conflict of interest must be provided	1
Quality of images (if applicable)	11a	Details of the equipment, software, and settings used to acquire the image(s) must be described in the text or legend	NA
	11b	The reason why the image(s) was acquired and the rationale for its inclusion in the manuscript must be provided in the text. A justification for all images which involve radiation must be included	NA
	11c	The circumstances (conditions) under which the image(s) were viewed and evaluated by the authors must be provided in the text	NA
	11d	The resolution and any magnification of the image(s) or any modifications/enhancements (e.g., adjustments for brightness, color balance, or magnification, image smoothing, staining, etc.) that were carried out must be described in the text or legend	NA
	11e	Patient(s) identifiers (names, patient numbers) must be removed to ensure they are anonymized	NA

 © 2025. The Author(s).

(*Continued*)

Section/topic	Item number	Checklist items	Reported on page number
	11f	An interpretation of the findings (meaning and implications) from the image(s) must be provided in the text	NA
	11g	The legend associated with each image must describe clearly what the subject is and what specific feature(s) it illustrates. Images of patients must describe the age, gender, and ethnicity of the person, if relevant	NA
	11h	Markers/labels must be used to identify the key information in the image(s) and defined in the legend	NA
	11i	The legend of each image must include an explanation whether it is pretreatment, intratreatment or posttreatment and, if relevant, how images were standardized over time	NA

Abbreviation: NA, not available.
[a]From: Nagendrababu V, Duncan HF, Bjørndal L, et al. PRIRATE 2020 guidelines for reporting randomized trials in endodontics: a consensus-based development. *Int End J* 2020. doi: 10.1111/iej.13294.
For further details visit: http://pride-endodonticguidelines.org/prirate/

 © 2025. The Author(s).
